# Supplementary material for: Recombinant FGF21 Attenuates Polychlorinated Biphenyl-Induced NAFLD/NASH by Modulating Hepatic Lipocalin-2 Expression
Source: Int J Mol Sci. 2022 Aug 10;23(16):8899. doi: 10.3390/ijms23168899 (PMC9408415; doi:10.3390/ijms23168899)
Supplement: Supplementary file 1 [file ijms-23-08899-s001.zip › ijms-1821103-supplementary.pdf]

Supplementary Materials for:

Recombinant FGF21 attenuates polychlorinated biphenyl-induced NAFLD/NASH by modulating hepatic lipocalin-2 expression

Hye Young Kim, and Young Hyun Yoo*

Department of Anatomy and Cell Biology and BK21 program, Department of Translational Biomedical Science, Dong-A University College of Medicine, Busan, Republic of Korea

Figures S1-3

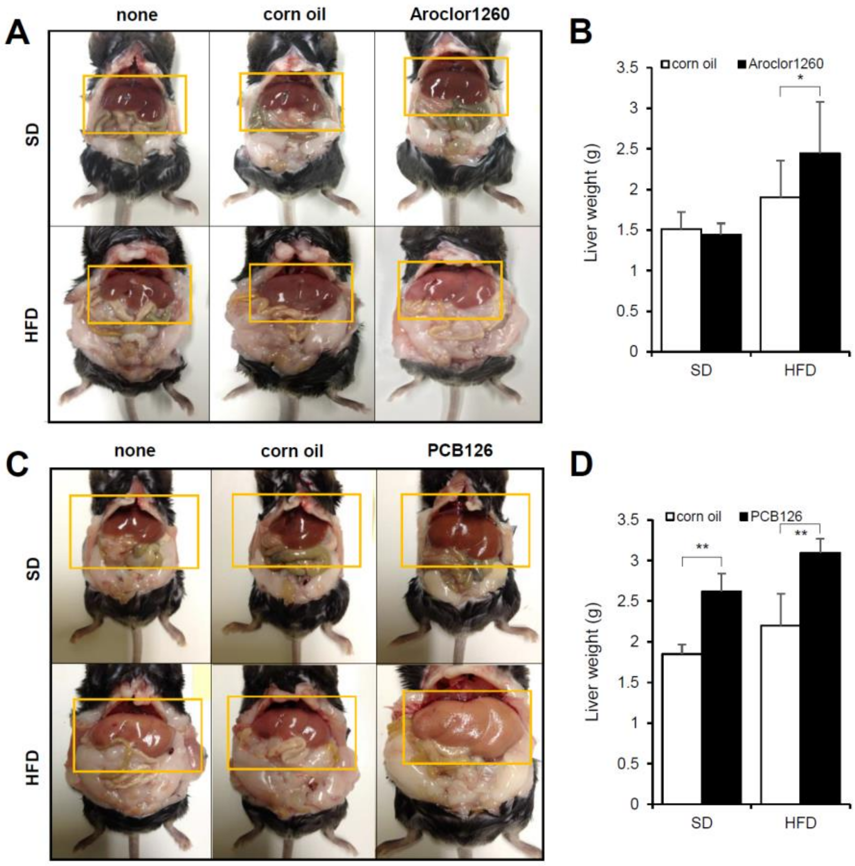

Figure S1. Aroclor1260 and PCB126 exposure induces NAFLD/NASH in C57BL/6 mice. Eight-week-old male C57BL/6 mice were fed either a SD or HFD for 4 weeks and then treated with vehicle (corn oil), Aroclor1260 (20 mg/kg) or PCB126 (5 mg/kg) by intraperitoneal injection for a total of four injections (2, 3, 4 and 5 weeks) during the 6-week study duration. (**A&C**) Representative photograph images of experimental animals. (**B&D**) Liver weight measurement. All values represent the mean±SD, n = 6-10 mice per group. *P < .05 and **P < .01.

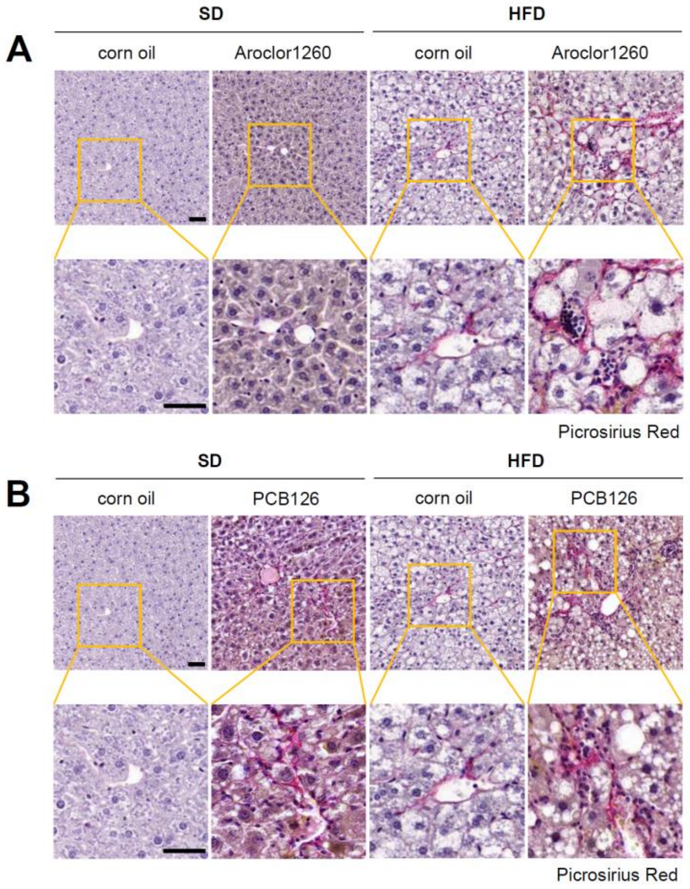

Figure S2. PCB exposure induces fibrosis in mice fed a SD or HFD. Eight-week-old male C57BL/6 mice were fed either a SD or HFD for 4 weeks and then treated with vehicle (corn oil), Aroclor1260 (20 mg/kg) or PCB126 (5 mg/kg) by intraperitoneal injection for a total of four injections (2, 3, 4 and 5 weeks) during the 6-week study duration. (**A&B**) Representative images of picrosirius red staining. Scale bar: 50 µm.

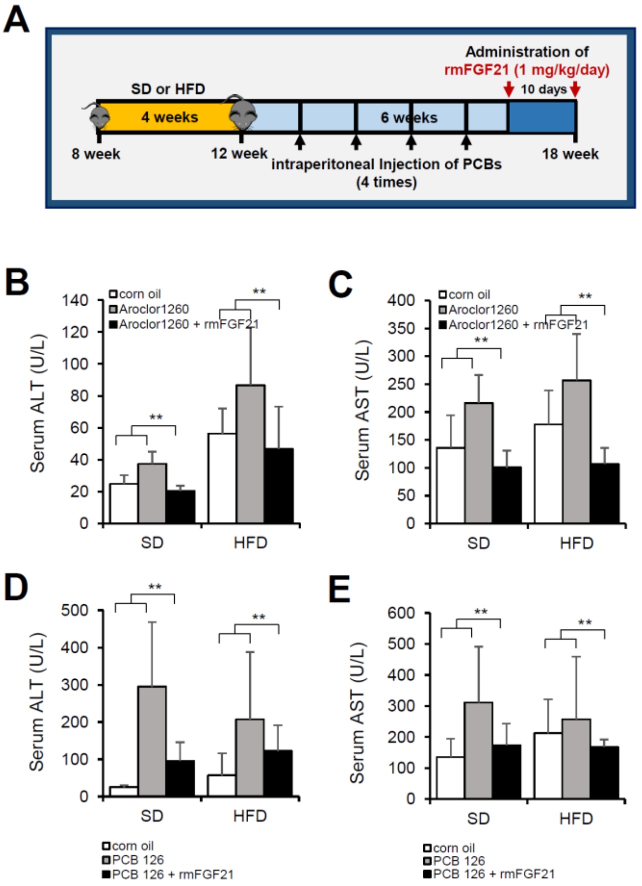

Figure S3. Recombinant FGF21 decreases PCB-induced liver injury in mice fed a SD or HFD. Eight-week-old male C57BL/6 mice were fed either a SD or HFD for 4 weeks and then treated with vehicle (corn oil), Aroclor1260 (20 mg/kg) or PCB126 (5 mg/kg) by intraperitoneal injection for a total of four injections (2, 3, 4 and 5 weeks) during the 6-week study duration. Aroclor1260- or PCB126-injected mice were intraperitoneally administered vehicle or rmFGF21 (1 mg/kg/day) once daily for 10 days. (**A**) Experimental design. (**B&D**) Serum ALT. (**C&E**) Serum AST. All values represent the mean±SD, n = 7-9 mice per group. $^{*}P < .05$ and $^{**}P < .01$.
